# The Estimated Temperature of the Semiconductor Diode Junction on the Basis of the Remote Thermographic Measurement

**DOI:** 10.3390/s23041944

**Published:** 2023-02-09

**Authors:** Arkadiusz Hulewicz, Krzysztof Dziarski, Zbigniew Krawiecki

**Affiliations:** 1 Institute of Electrical Engineering and Electronics, Poznan University of Technology, Piotrowo 3A, 60-965 Poznan, Poland; 2 Institute of Electric Power Engineering, Poznan University of Technology, Piotrowo 3A, 60-965 Poznan, Poland

**Keywords:** diode, LabVIEW, semiconductor die, silicon carbide, SolidWorks, thermography

## Abstract

The value of a semiconductor’s diode temperature determines the correct operation of this element and its useful lifetime. One of the methods for determining the die temperature of a semiconductor diode is through the use of indirect thermographic measurements. The accuracy of the thermographic temperature measurement of the diode case depends on the prevailing conditions. The temperature of the mold body (the black part of the diode case made of epoxy resin) depends on the place of measurement. The temperature of the place above the die is closer to the die temperature than the temperature of mold body fragments above the base plate. In addition, the difficulty of its thermographic temperature measurement increases when the surface whose temperature is being measured is in motion. Then, the temperature measured by thermography may not apply to the warmest point in the case where the die temperature is determined. Information about the difference between temperatures of the different parts of the mold body and the die may be important. For this reason, it was decided to check how much the temperature measurement error of the die diode changes if the temperature of the diode case is not measured at the point that is above the die.

## 1. Introduction

Some components mounted on printed circuit boards (PCBs) are semiconductor components. Semiconductor components are diodes and transistors. The correct operation of other elements placed on the PCB and, consequently, of the entire device depends on its proper operation [1]. Examples of devices in which semiconductor elements are used are AC/DC and DC/AC converters used in electromobility and renewable energy. Another example is devices that work as nodes on the Internet of Things (IoT) [2].

A semiconductor diode is an example of a semiconductor component. It is a single junction between two p-enriched (positive) and n-depleted (negative) regions. The p–n junction is placed in an epoxy mold compound. One area is connected to the lead frame with a fine wire less than 1 mm thick [3]. In the case of the TO-220 enclosure, the second area is connected to the metal back metal case. In turn, this part of the case is connected to the lead frame [4].

A semiconductor diode is a single junction between p-type and n-type semiconductors (mostly silicon) [3,4]. The correct operation of a semiconductor diode depends on the temperature of the semiconductor junction *T_j_*. When the value of *T_j_* is higher than the maximum value of *T_jmax_*, the semiconductor junction will be damaged. The operation of the diode with an excessive value of *Tj* (*T_j_* < *T_jmaxc_*) shortens the life of the diode. Another effect of the operation of the semiconductor diode junction with *T_j_* different from the value taken into account during the design process is a change in the characteristics that link the value of forward voltage *V_F_* and forward current *I_F_* (*I_F_* = f(*V_F_*)) [5]. It changes compared to the characteristics taken into account during the design process. As a consequence, a change in the *T_j_* value changes the *V_F_* and *I_F_* values of the semiconductor junction [6].

Obtaining a stable semiconductor junction temperature is possible after using a cooling system. This is associated with additional costs. Oversized cooling systems increase the costs of cooling the semiconductor junction. However, when the selected cooling system dissipates heat incorrectly, the *T_j_* value increases to excessively high values. Therefore, to verify the correct selection of the cooling system (e.g., heat sink), the value of *T_j_* should be known [7].

There are several groups of methods that allow for estimation the *T_j_* value. Direct methods belong to the first group of methods. The second group of methods is indirect methods. Direct methods can be divided into contact methods and noncontact methods. Indirect methods can be divided into contact, noncontact and electrical methods.

Direct methods include those that require access to the semiconductor junction. Direct contact methods rely on the application of a temperature sensor directly to a semiconductor junction. Noncontact direct methods rely on estimating the temperature of a semiconductor junction on the basis of optical radiation emitted by the junction [8]. It is also possible to estimate the junction temperature on the basis of the angle of reflection of the optical radiation from the semiconductor element [9].

The disadvantage of these methods is their necessity to destroy the case. It is an irreversible process. As a result of case destruction, the heat exchange between the junction and the environment changes. The distribution of thermal resistances on the junction–ambient path changes. For this reason, the temperature of the junction operating in the open case and the junction operating in the closed case is different. This is noticeable even when the same power *P_j_* is dissipated at the junction.

The indirect methods do not require direct access to the semiconductor junction. They rely on estimating the temperature of the semiconductor junction on the basis of the temperature of the case. The temperature of the case can be measured with an applied temperature sensor. The disadvantage of this method is the unknown value of the thermal resistance between the junction and the case [10]. Applying the sensor to the case can change the temperature distribution on its surface. Another disadvantage of this method is the risk of applying a metal temperature sensor to the metal part of the case which may, consequently, result in an electric shock [11,12].

Electrical methods rely on determining the temperature of a semiconductor junction on the basis of a known value of an electrical parameter, the value of which depends on the temperature of the junction. The parameter is called the *TSP* (thermal sensitive parameter). For a semiconductor diode, *TSP* is *V_F_*. In order to determine the semiconductor junction temperature using this method, the characteristic *T_j_* = f(*TSP*) must be known. It is not possible to determine identical characteristics for different junctions [13]. This is because of the impossibility of growing two identical semiconductor crystals. Furthermore, to determine the characteristic *T_j_* = f(*TSP*), additional research should be performed. For this reason, the use of this method is awkward [14].

These inconveniences can be prevented by using thermography. This noncontact method allows for measuring the junction temperature on the basis of the power of IR radiation (InfraRed) reaching the IR radiation detectors [15]. With the radiation emitted by the observed case, the lens of the IR camera is reached by the radiation from neighboring objects and radiation emitted by the air layer located between the lens and the case of the electronic component. For this reason, during the thermographic measurement of the temperature of the case, the conditions that prevail during the measurement should be taken into account.

The most important of them include the value of the emissivity coefficient *ε* [16], the reflected temperature *T_r_* [17], the distance between the lens and the observed object *d* [18], the ambient temperature *T_a_* [19], the temperature of the external optical system [20], the transmittance of the external optical system [21,22], the relative humidity *ω* [23], the viewing angle *β* [24] and the sharpness of the recorded thermogram [25].

The case of semiconductor elements is made of various materials. Regarding TO cases (e.g., TO-247), the case consists of a copper plate on which a die is placed. The die is connected to the leads by bond wire and copper plate. The die is embedded in a mold body that is made of an EMC (epoxy molding compound). Case fragments have different values of *ε*. The back part of the case, which is made of bleached copper plate, has a lower *ε* value compared to the mold body. The closest to the die is the copper plate and the part of the mold body that is placed directly above the die. Metals have a high reflectance *ρ*. For this reason, the lens of the thermographic camera, in addition to the temperature of the copper plate, receives a large amount of IR radiation, which comes from neighboring objects. This feature makes thermographic measurements difficult in this spot.

The semiconductor elements are made of various materials. One of them is silicon carbide (SiC). Compared to silicon Si, SiC has a higher *T_jmax_* value. Elements made on the basis of SiC are used in the areas of renewable energy and electromobility. For this reason, it was decided to carry out research work aimed at determining the junction temperature of a semiconductor diode made on the basis of SiC.

At the prototyping stage, the value of the junction temperature of the diode placed on the heat sink is important. This allows for the assessment of the operating point of the element and the correctness of heat sink selections. As a consequence, it is possible to minimize costs. The prototyping time is also important. The cases of the electronic components whose temperature is determined are usually small in size. Therefore, a precise measurement of the case temperature with a thermographic camera is required, which results in extending the useful life of the case.

Information about the differences between the temperature of the part of the mold body, which are above the die and the temperature of the part of the mold body, which are above the base plate, are unknown to the authors of this article. Articles about these differences are unknown to the authors, too. This information is difficult to find in the literature.

For this reason, it was decided to use the methods that would allow the remote execution of such a thermogram, on the basis of which it would be possible to estimate the junction temperature for a semiconductor diode. It was decided to check how the complexity of the applied algorithm affects the time of determining the die temperature. How much the die temperature determined on the basis of the maximum case temperature differs from the die temperature determined on the basis of the case temperature of another part of the mold body was also checked.

## 2. Materials and Methods

### 2.1. Measurement System

The temperature of the diode case *T_c_* is dependent on the junction temperature *T_j_*. In turn, *T_j_* depends on the power dissipated in the junction *P_j_*. A silicon carbide Schottky diode, FFSH10120A (Onsemi, Phoenix, AZ, USA) (*I_F_* = 10 A, *V_F_* = 1.5 V), placed in a TO-247 case was selected for this research. The view of the diode and the external dimensions of the TO-247 case are shown in Refs. [26,27].

The value of *P_j_* was determined on the basis of the measurements performed, as the product of *I_F_*, the measured voltage drop at the diode terminals *V_D_*, and the cosine of the phase shift cosφ between *I_D_* and *V_D_*. *I_D_* and *V_D_* values were measured with a UT51 multimeter manual (UNI-T, Dongguan City, China).

The current measurement error *ΔI_D_* and the voltage measurement error *ΔV_D_* were calculated according to the UT51 multimeter manual. Calculating the values of *ΔI_D_* and *ΔV_D_* made it possible to determine the error value of the junction power measurement *ΔP_j_* in accordance with Equation (1) [28]:

(1) $$\Delta P_{j}=\Delta I_{D}\cdot V_{D}+\Delta V_{D}\cdot I_{D}$$

**Figure 1:**
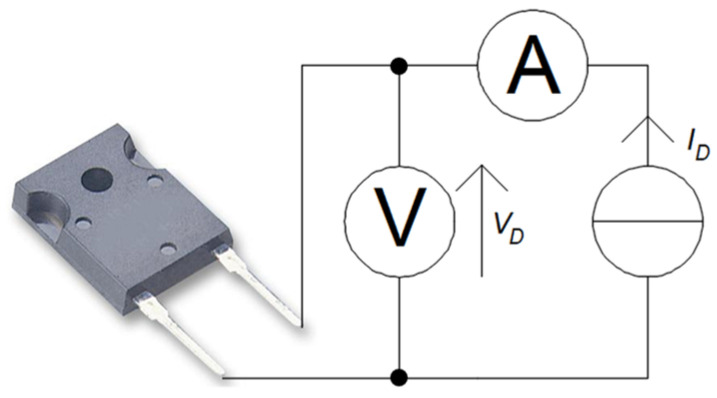
Measuring system used to measure the power dissipated at the *P_j_* junction.

The measuring system that was used to measure the *P_j_* value is shown in Figure 1.

**Figure 2:**
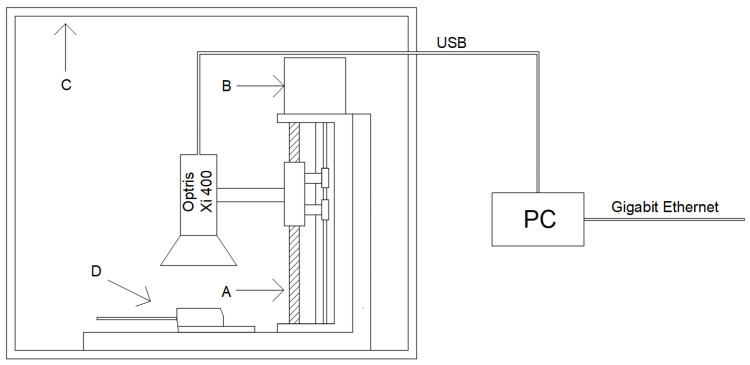
Measurement system with Optris Xi400 camera and FFSH10120A diode: (**A**) tripod; (**B**) stepper motor; (**C**) chamber made of black foam made of polyurethane; (**D**) the observed semiconductor element.

The case of the FFSH10120A diode that operates in the circuit shown in Figure 2 was observed with an Optris Xi 400 camera (Optris, Berlin, Germany). The Xi 400 model is equipped with a matrix with a spatial resolution of 382x288 pixels and a microscope lens (focal length *F* = 20 mm, minimum distance from the measured object *WD_min_* = 0.35 m, *IFOV* (*instantaneous field of view*) = 0.9 mrad). The camera was connected to the computer via USB interface. The computer was equipped with the Windows 10 operating system, Intel Core i5-6500T processor and 16 GB RAM. The computer was connected to the Internet via gigabit ethernet. Optris PIX Connect software (Optris, Berlin, Germany) was installed on the computer to enable thermograms. The thermograms were sent via an Internet connection to computers with Optris PIX Connect software, SolidWorks 2022 (Dassault Systèmes, Vélizy-Villacoublay, France) and LabVIEW 2022 (National Instruments, Austin, TX, USA) installed.

The camera and diode were placed in a plexiglass chamber. The external dimensions of the chamber were 45 × 35 × 35 cm, while the internal dimensions of the chamber were 40 × 30 × 30 cm. The chamber restricts air exchange with the environment. The walls of the chamber do not allow visible light to pass through. The chamber walls were lined with black foam made of polyurethane. The foam used is characterized by a porous structure, and every single pore of the foam resembles the black body cavity model. The walls of the chamber walls so prepared are characterized by a high value of the emissivity factor *ε =* 0.95 [29] and a small value of the reflectance factor *ρ*. The measurement system prepared in this way is shown in Figure 2.

**Figure 3:**
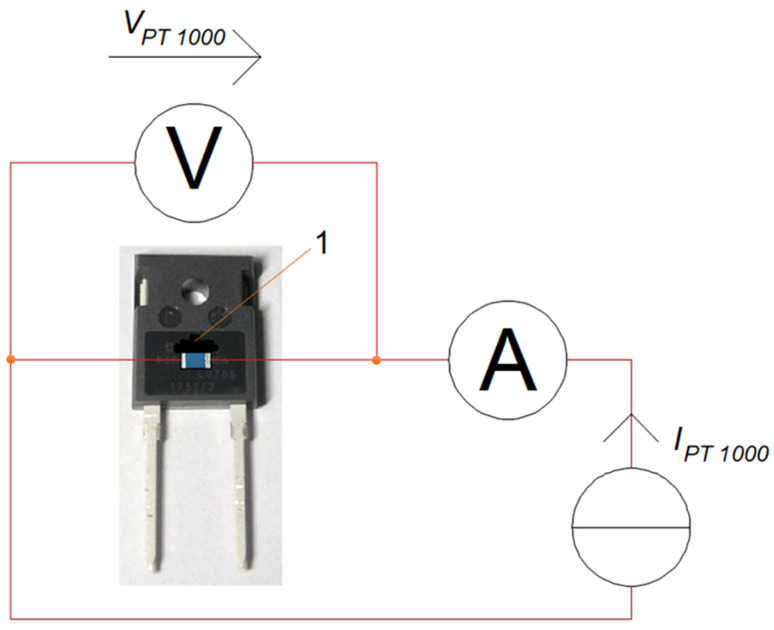
The spot to place the PT 1000 sensor on the mold body of the FFSH10120A diode and the marker made of Velvet Coating 811-21 paint—point 1.

The measurement system prepared in this way made it possible to carry out research work, the result of which was the determination of the value of *ε* of the mold body [30]. For this purpose, a PT 1000 temperature sensor placed in an SMD 0603 case (Heraeus, Hanau, Germany) [31] was attached to the mold body of the FFSH10120A diode case [32]. The sensor was glued to the diode case. The WLK 5 glue (Fischer Elektronik, Lüdenscheid, Germany) with a known value of thermal conductivity *k* (*k* = 0.836 W/m·K) was used to stick the sensor to the case. The mold body was marked with a marker made of Velvet Coating 811-21 paint with a known emissivity coefficient value e ranging from 0.970 to 0.975 for temperatures within the limit of −36 °C to 82 °C. The uncertainty with which the value of the emissivity coefficient value was determined was 0.004. Figure 3 shows the placement of the PT 1000 sensor and the Velvet Coating 811-21 paint marker.

**Figure 4:**
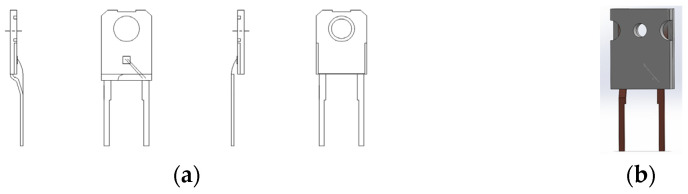
(**a**) Internal view of the TO-247-2 case with the FFSH10120A diode; (**b**) FFSH10120A diode model made in SolidWorks.

The known value of *ε* was entered into the thermographic camera. The temperature was then measured on the *T_cM_* marker (point 1 in Figure 4). The temperature measured on the marker was assumed to be the same as the temperature of the part of the mold body not covered with the *T_c_* paint, which is located directly next to it. The camera lens was focused on the mold body fragment located right next to the marker. The value of *ε* was changed until the value of *T_c_* was the same as the value of *T_cM_*. The value of *ε* for which the *T_c_* values are equal to *T_cM_* is taken as *ε* of the mold body.

### 2.2. Simulations of Temperature Distribution in the TO-247 Case

In order to perform the correct simulations of the temperature distribution inside the TO-247 case, it was necessary to accurately reproduce the internal dimensions of the case. For this purpose, a mold body made of EMC (epoxy molding compound) was removed from the case. The internal dimensions were measured using a Motic Images Plus 3.0 microscope (*Motic*, Hong Kong, China). The internal view of the case of the FFSH10120A diode and the model made in SolidWorks are shown in Figure 4.

Heat transfer in the x direction between two points can be described by Equation (2) [33]:

(2) $$-q_{v}=k\cdot \frac{\partial^{2}T}{\partial^{2}x}-C_{th}\cdot \frac{\partial T}{\partial x}$$

where: *q_v_* is the power density dissipated in the die in W/m^3^, *C_th_* is the thermal capacity, and *T* is the temperature.

In steady state, Equation (2) takes the form of Equation (3) [34,35].

(3) $$q=k\cdot \frac{\partial T}{\partial x}$$

where *q* is the heat flux in W/m^2^, and *k* is the thermal conductivity J/Km^3^.

After separating the variables and integrating Equation (3) on both sides, the time constant can be obtained from the following boundary conditions (Equation (4)):

for *x* = 0→*T* = *T*_1_
for *x* = *xk*→*T* = *T*_2_ (4)

After determining the time constant, when *q* penetrates the entire wall, Equation (3) takes the form of Equation (5):

(5) $$T_{1}-T_{2}=\frac{P_{c}}{S\cdot k}\cdot x$$

where *P_c_* is the total power applied to the wall (W) and *S* (m^2^) is the area of the wall penetrated by *J* (W∙m^−2^).

Determining the temperature difference using Equation (5) is simple in the case of determining 1D models (the temperature difference is determined in one line). In the case of determining the temperature distribution in a solid (3D models), the number of calculations increases. The more complicated the shape of the solid, the more the number of calculations needed to determine the temperature distribution in the solid increases. In this case, numerical methods are used to determine the temperature distribution inside the solid. One of them is the finite element method (FEM). Another one of them is the FEA (finite element analysis) method. By definition, FEA is a numerical method for solving problems in engineering and mathematical physics [36].

In FEM, the area in which the temperature distribution is sought is divided into a finite number of tetrahedral elements. In each of the tetrahedral elements, the temperature field is interpolated based on the temperature in the nodes of this element and the linear shape functions are determined using Equation (6) [12]:

(6) $$T\left(x,y,z,t\right)=\sum_{i=1}^{4}H_{i}\cdot T_{i}\left(t\right)$$

where *T_i_*(*t*) is the nodal temperature at node *i*, *H_i_* is the linear shape function.

In the case of a Cartesian coordinate system, the linear temperature function (Equation (6)) for node *i* (in a tetrahedral element) can be expressed as Equation (7) [37]:

(7) $$N_{i}\left(x,y,z\right)=a_{i}+b_{i}\cdot x+c_{i}\cdot y+d_{i}\cdot z$$

where *i* = 1,…,4., *a_i_*, *b_i_*, *c_i_* and *d_i_*—the coefficients.

Consequently, a system of equations can be obtained for the unknown coefficients. This procedure should be repeated for all grid points. After the shape function is derived and integrated, a discrete system of equations can be obtained. When the direction of the edge of a tetrahedral element does not coincide with the direction of any axis of the coordinate system, the calculation becomes more complicated. In this case, each point, *x*, *y* and *z* of the original coordinate system should be changed to another point (*ξ*, *η*, *ζ*) in the transformed coordinate system, whose axis directions are consistent with the edge of the tetrahedral element—Equation (8) [38]:

(8) $$\begin{array}{c} x=x_{1}+\left(x_{2}-x_{1}\right)\cdot \xi +\left(x_{3}-x_{1}\right)\cdot \eta +\left(x_{4}-x_{1}\right)\cdot \zeta \\ y=y_{1}+\left(y_{2}-y_{1}\right)\cdot \xi +\left(y_{3}-y_{1}\right)\cdot \eta +\left(y_{4}-y_{1}\right)\cdot \zeta \\ z=z_{1}+\left(z_{2}-z_{1}\right)\cdot \xi +\left(z_{3}-z_{1}\right)\cdot \eta +\left(z_{4}-z_{1}\right)\cdot \zeta \end{array}$$

The equations *J* of the Jacobi matrix can be described by Equation (9):

(9) $$J=\left[\begin{array}{ccc} x_{2}-x_{1} & x_{3}-x_{1} & x_{4}-x_{1}\\ y_{2}-y_{1} & y_{3}-y_{1} & y_{4}-y_{1}\\ z_{2}-z_{1} & z_{3}-z_{1} & z_{4}-z_{1} \end{array}\right]$$

Shape functions for the transformed coordinate system can be written using Equation (10) [38]:

(10) $$\begin{array}{c} N_{1}\left(\xi ,\;\eta ,\;\zeta \right)=1-\xi -\eta -\zeta \\ N_{2}\left(\xi ,\;\eta ,\;\zeta \right)=\xi \\ N_{3}\left(\xi ,\;\eta ,\;\zeta \right)=\eta \\ N_{4}\left(\xi ,\;\eta ,\;\zeta \right)=\zeta \end{array}$$

Performing correct simulations of the temperature distribution in the case of a semiconductor element requires determining the amount of heat emitted by radiation (through thermal radiation) and released by convection. The amount of heat given off by the radiation per unit of time per unit of area is determined by the radiation coefficient *h_r_*. The value of the radiation coefficient can be obtained using Equation (11) [39]:

(11) $$h_{r}=\varepsilon \cdot \sigma_{c}\cdot \left(T_{c}+T_{a}\right)\cdot \left(T_{c}^{2}+T_{a}^{2}\right)$$

where *σ_c_* is the Stefan–Boltzmann constant equal to 5.67 × 10^−8^ (W∙m^−2^∙K^−4^)), *T_c_* is the case temperature (K) and *T_a_* is the air temperature (K).

The value of the convection coefficient *h_c_* determines the amount of heat released by the convection per unit of time per unit of area. The *h_c_* value is difficult to determine, as it depends, among other things, on the shape, complexity and temperature of the object that gives off heat by convection. The *h_c_* coefficient was selected using the theory of similarity to physical phenomena. Relationships with physical quantities characterizing a given phenomenon were described using the criteria of Nusselt, Grashof and Prandtl. When determining the convection coefficient for a flat surface, Equation (12) [40] is used:

(12) $$h_{c}=\frac{Nu\cdot k}{L}$$

where *h_c_* is the convection coefficient of flat surfaces, *Nu* is the Nusselt number (−) and *L* is the characteristic length in meters (for a vertical wall, it is its height).

The Nusselt number can be expressed using Equation (13) [41]:

(13) $$Nu=a\cdot {\left(Gr\cdot Pr\right)}^{b}$$

where *a* and *b* are dimensionless coefficients, the values of which depend on the shape and orientation of the analyzed surface and the product *Pr*·*Gr*. *Pr* (−) is the Prandtl numer, *Gr* is the Grashof number.

The values of the coefficients *a* and *b* depend on the product of the numbers *G_r_* and *P_r_* of the orientation of the surface for which the *h_c_* value is calculated and the way the air flows around the surface (laminar flow, turbulent flow). The values of the coefficients *a* and *b* can be read in Table 1.

The Prandtl number can be obtained using Equation (14) [18]:

(14) $$P_{r}=\frac{c\cdot \eta }{k}$$

where *c* is the specific air heat equal to 1005 (J∙kg^−1^∙K^−1^) in 293.15 (K), *η* is the dynamic air viscosity equal to 1.75 × 10^−5^ (kg∙m^−1^∙s^−1^) in 273.15 (K).

Grashof’s number is obtained from Equation (15) [42]:

(15) $$G_{r}=\frac{\alpha \cdot g\cdot \left(T_{S}-T_{a}\right)\cdot \rho^{2}\cdot L^{3}}{\eta^{2}}$$

where *α* is a coefficient of expansion equal to 0.0034 (K^−1^), *g* is the gravitational acceleration of 9.8 (m∙s^−2^), *ρ* is air density equal to 1.21 (kg∙m^−3^) in 273.15 (K).

In the event that the velocity of the air around a semiconductor case is faster than 0.5 m/s (e.g., due to the use of a fan), it is necessary to calculate the Nusselt number with another criteria number, and that is the Reynolds number. In this research, only natural convection was considered. For this reason, the authors refrained from describing the calculation of the Reynolds and Nusselt numbers for forced convection. The presented methods for obtaining the convection coefficient are approximate methods. In order to obtain a better factor, it is necessary to carry out further research.

### 2.3. Image Processing in LabVIEW

Thermographs obtained with the Optris Xi400 thermographic camera (Optris, Berlin, Germany) were analyzed. First, they were digitally processed. This made it possible to sharpen the edges of the observed semiconductor element. It was also possible to indicate a point on the mold body (the black part of the case) where the highest temperature was recorded. According to the assumptions, the temperature value in this place should be as close to the die temperature as possible. For this purpose, an image with a resolution of 382 × 288 pixels and color depth of 24 bits was obtained from a thermographic camera. To process such an image, a program was developed in the graphic programming environment LabVIEW by using National Instruments with the IMAQ package [43,44].

The created program is adapted to process files of typical formats such as BMP, TIFF, JPEG, PNG, etc. LabVIEW, using the IMAQ Create library, creates a temporary location in the computer’s memory for image processing. After the image is loaded, the pixels are automatically converted to the format set by the user. The paper presents the results after the conversion to a monochrome unsigned 8-bit format. The pixel color obtained in this way ranges from 0 to 255, with 0 being black and 255 being white. The image created as a result of the conversion is the so-called reference image [45,46].

The reference image is provided to subsequent subprograms (subVIs) that are in the code of the main program to process the information stored in it. The reference image then is visualized. In addition, it is also converted to a matrix in which pixel color values are entered. The data contained in the pixel matrix is used to create a histogram. The histogram shows the number of pixels in a given grayscale color. The program uses the function of extracting a fragment from an image selected by the user. This can be achieved by using the mouse cursor or by defining an array of four coordinates of the cut area. This functionality was introduced to avoid misinterpretation of the temperature of neighboring areas. In the observation field of the camera, there are materials with different emissivity coefficients. The emissivity coefficient set in the camera menu corresponds to the surface of the diode case. The temperature read for neighboring materials may not be correct. The result may be underestimated or overestimated. The area extracted from the entire image is then trimmed to narrow the set of hot pixels.

**Figure 5:**
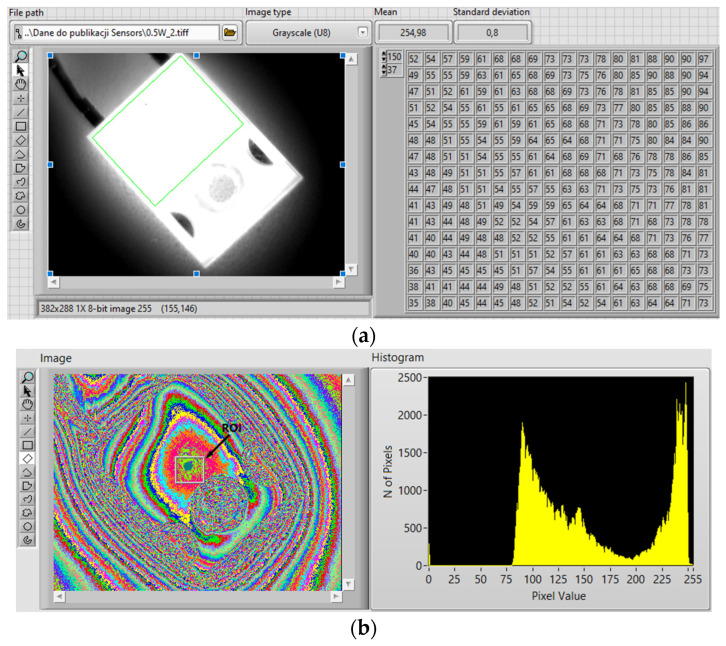
(**a**) A panel of the executed program with the sample results of the processing image; (**b**) a true grayscale image and its histogram.

Using thresholding, the region of interest (ROI) with the highest temperature is designated. The color values of this area are presented in the next histogram. In addition, the average color value, the standard deviation, the minimum and maximum values for this area are calculated and displayed. The given values indirectly determine the temperature distribution around the mean value; the larger the standard deviation, the more dispersed the data is, and the more diverse the area is too. Figure 5a shows the panel of the executed program with the results of processing an example image from a thermographic camera. Figure 5b shows the converted image with real grayscale and its histogram (the gray rectangle is the ROI with the highest temperature, and the green point is the spot with the maximum temperature of the object).

## 3. Results

Case thermograms used in the research were made in the measuring system shown in Figure 3. In such a system, the camera was placed at a distance of 300 mm from the tested object. The thermographs were acquired remotely using Optris PIX Connect software. The obtained thermograms were subjected to further mathematical analysis. SolidWorks and LabVIEW software were used for this purpose. In addition to precise dimensioning of the tested object, thermal simulations in SolidWorks software required knowledge of the *h_c_*, *ε* coefficients and the power dissipated in the die. The *h_c_* coefficients were determined on the basis of Equations (12)–(15). The value of *ε* was determined experimentally, and the values of *P_j_* were determined on the basis of the product of the measured *I_F_* and the voltage drop across the terminals of the diode *V_D_*. The values of *ΔP_j_* were determined on the basis of the Equation 1 and the values of *ΔI_D_* and *ΔU_D_* were determined based on the instruction UT51 [47]. The results obtained are presented in Table 2.

The values of the other coefficients related to the materials used for the construction of the diode are presented in Table 3. The parameters of these coefficients are necessary for performing thermal simulations.

**Figure 6:**
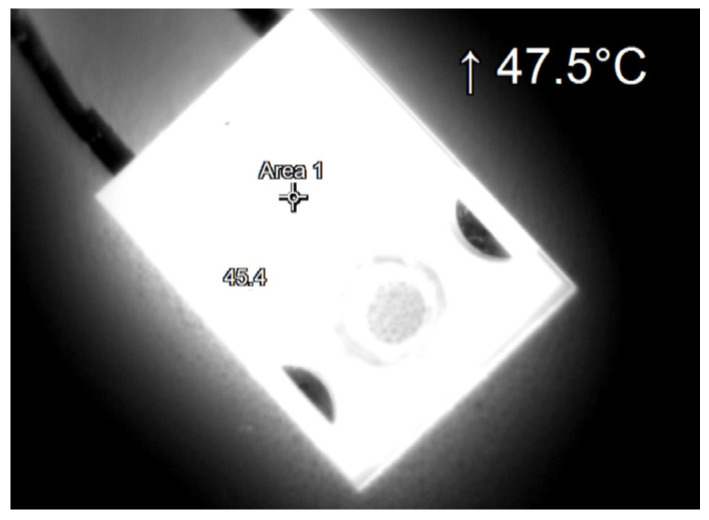
The case thermogram of the FFSH10120A diode. The average temperature at the cursor: 45 °C. The maximum temperature at the cursor (the area is equal to 9xIFOV (instantaneous field of view)) is 47.5 °C. The power dissipated at the junction *P_j_* = 0.57 W.

**Figure 7:**
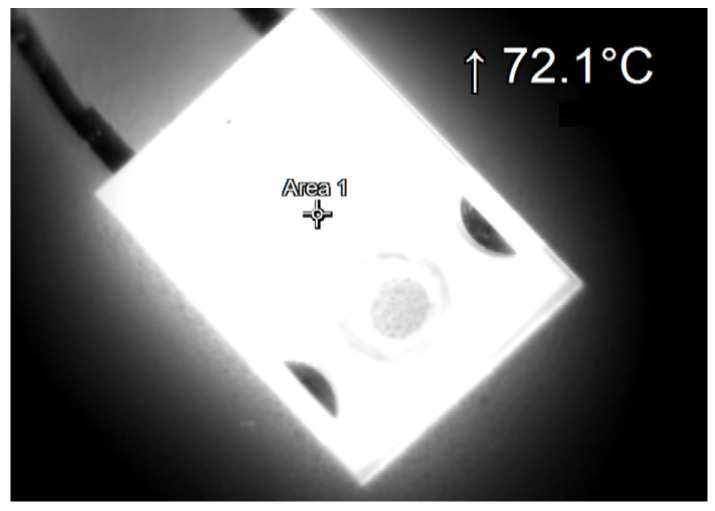
The case thermogram of the FFSH10120A diode. The average temperature at the cursor: 70.4 °C. The maximum temperature at the cursor (the area is equal to 9xIFOV (instantaneous field of view)) is 72.1 °C. The power dissipated at the junction *P_j_* = 1.32 W.

**Figure 8:**
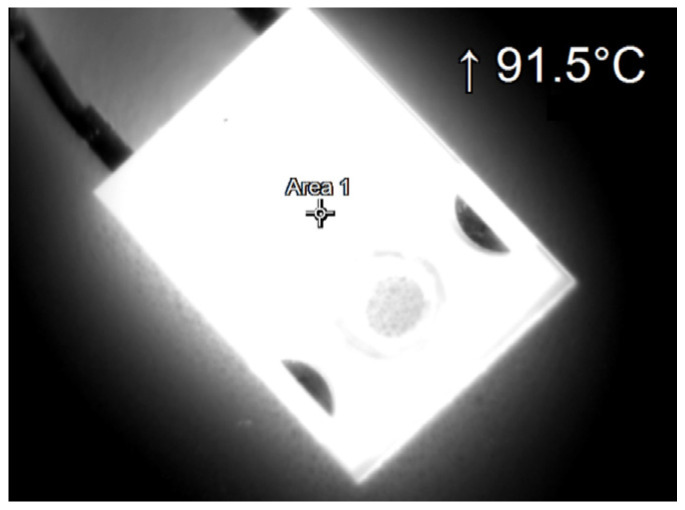
The case thermogram of the FFSH10120A diode. The average temperature at the cursor: 89.6 °C. The maximum temperature at the cursor (the area is equal to 9xIFOV (instantaneous field of view)) is 91.5 °C. The power dissipated at the junction *P_j_* = 1.71 W.

**Figure 9:**
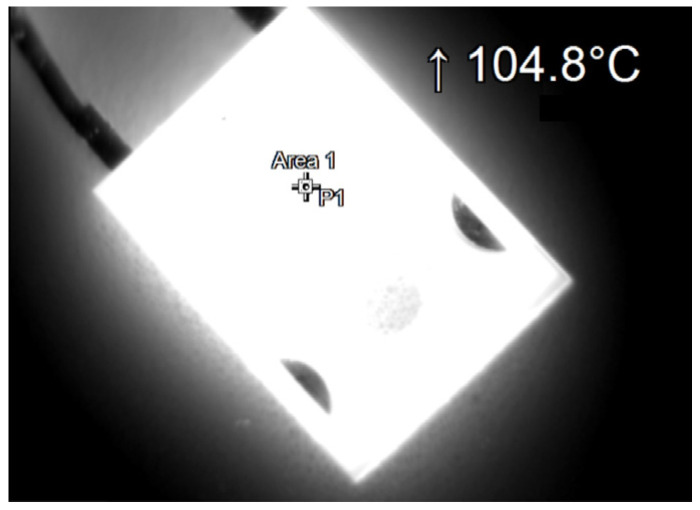
The case thermogram of the FFSH10120A diode. The average temperature at the cursor: 102.4 °C. The maximum temperature at the cursor (the area is equal to 9xIFOV (instantaneous field of view)) is 104.8 °C. The power dissipated at the junction *P_j_* = 2.19 W.

**Figure 10:**
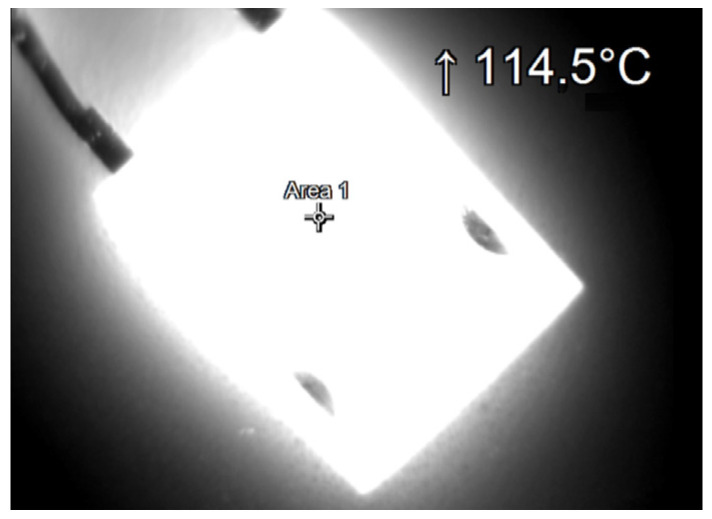
The case thermogram of the FFSH10120A diode. The average temperature at the cursor: 112.2 °C. The maximum temperature at the cursor (the area is equal to 9xIFOV (instantaneous field of view)) is 114.5 °C. The power dissipated at the junction *P_j_* = 2.55 W.

**Figure 11:**
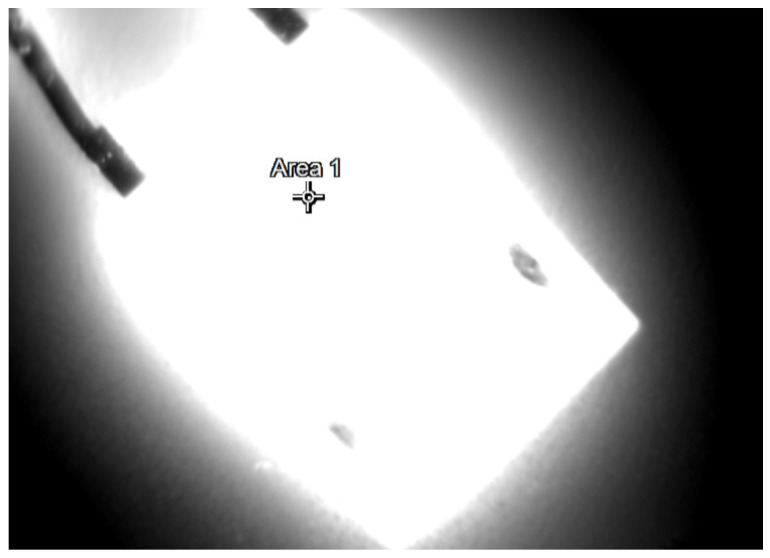
The case thermogram of the FFSH10120A diode. The average temperature at the cursor: 123.8 °C. The maximum temperature at the cursor (the area is equal to 9xIFOV (instantaneous field of view)) 125.4 °C. The power dissipated at the junction *P_j_* = 3.01 W.

During the tests, thermographic measurements of the diode case were made for the several selected values of the power *P_j_* dissipated in the die. The thermograms recorded during the measurements are shown in Figure 6, Figure 7, Figure 8, Figure 9, Figure 10 and Figure 11.

**Figure 12:**
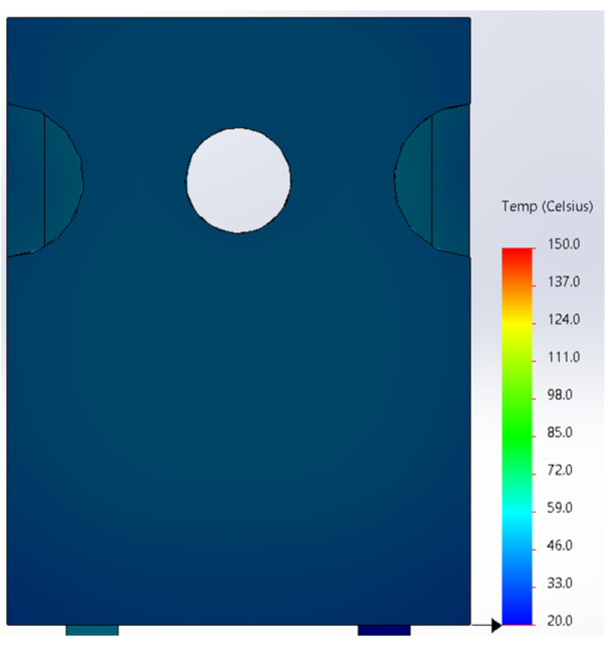
The simulation result for the case of the FFSH10120A diode. The maximum recorded temperature: 47.2 °C. The power dissipated at the junction *P_j_* = 0.57 W. The temperature ranges from 20 °C to 150 °C.

**Figure 13:**
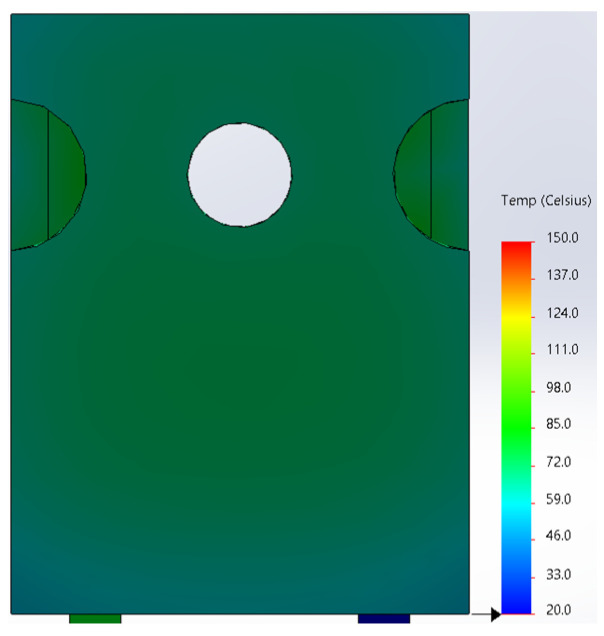
The simulation result for the case of the FFSH10120A diode. The maximum recorded temperature: 72.5 °C. The power dissipated at the junction *P_j_* = 1.32 W. The temperature ranges from 20 °C to 150 °C.

**Figure 14:**
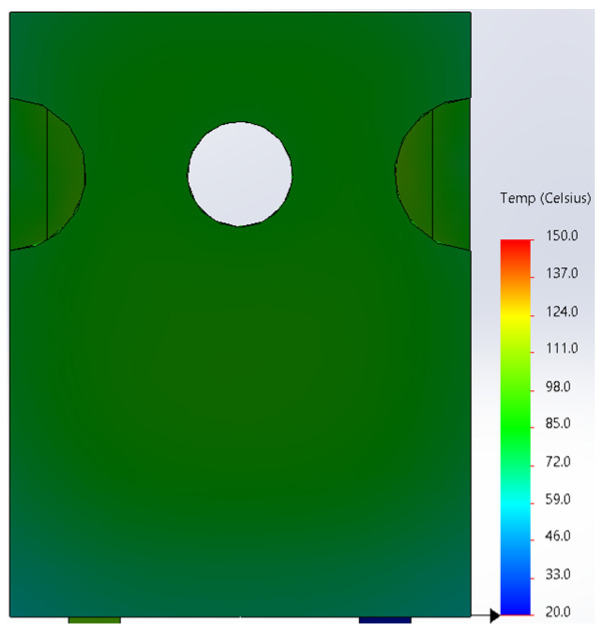
The simulation result for the case of the FFSH10120A diode. The maximum recorded temperature: 90.6 °C. The power dissipated at the junction *P_j_* = 1.71 W. The temperature ranges from 20 °C to 150 °C.

**Figure 15:**
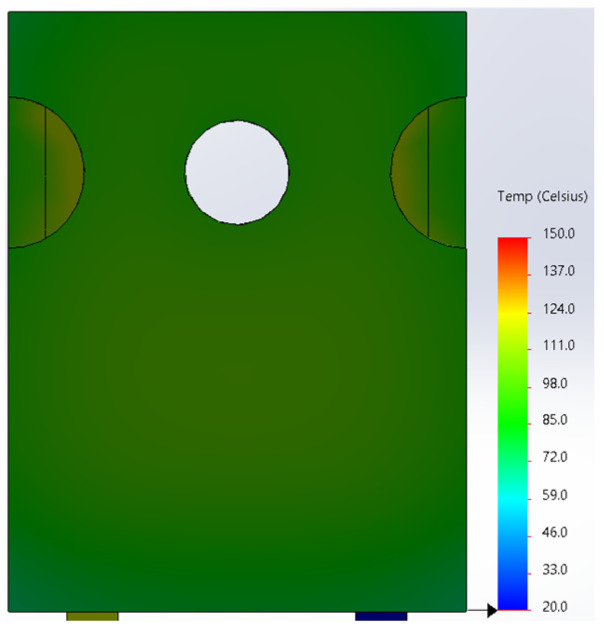
The simulation result for the case of the FFSH10120A diode. The maximum recorded temperature: 103.6 °C. The power dissipated at the junction *P_j_* = 2.19 W. The temperature ranges from 20 °C to 150 °C.

**Figure 16:**
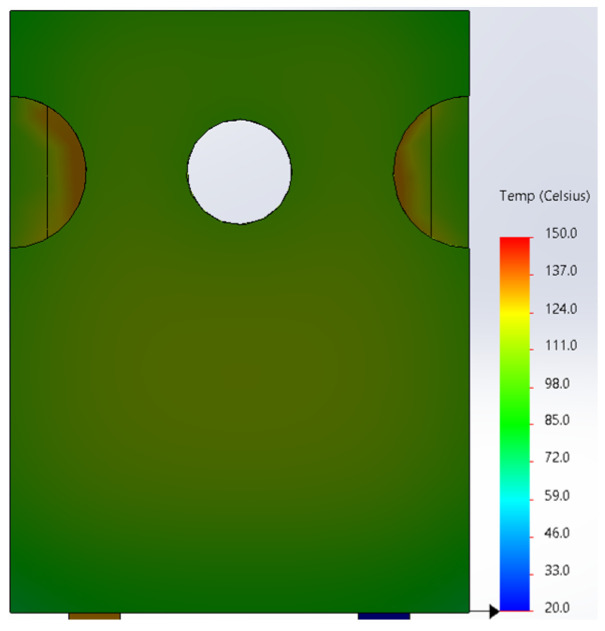
The simulation result for the case of the FFSH10120A diode. The maximum recorded temperature: 115.0 °C. The power dissipated at the junction *P_j_* = 2.55 W. The temperature ranges from 20 °C to 150 °C.

**Figure 17:**
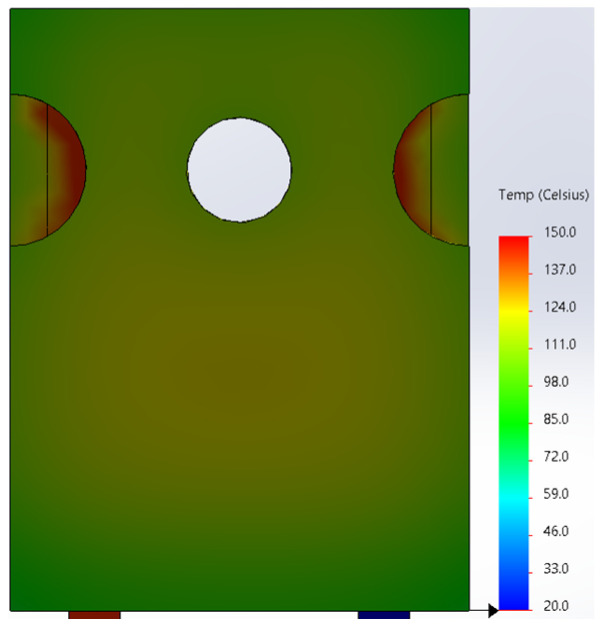
The simulation result for the case of the FFSH10120A diode. The maximum recorded temperature: 125.0 °C. The power dissipated at the junction *P_j_* = 3.01 W. The temperature ranges from 20 °C to 150 °C.

Based on the knowledge of the parameters related to the construction of the tested diode, its model was developed in SolidWorks. On this basis, the thermal simulations were performed for all values of the power dissipated in the die *P_j_*, for which the thermographic measurements were taken earlier. The temperature distributions obtained as a result of the simulations are shown in Figure 12, Figure 13, Figure 14, Figure 15, Figure 16 and Figure 17.

**Figure 18:**
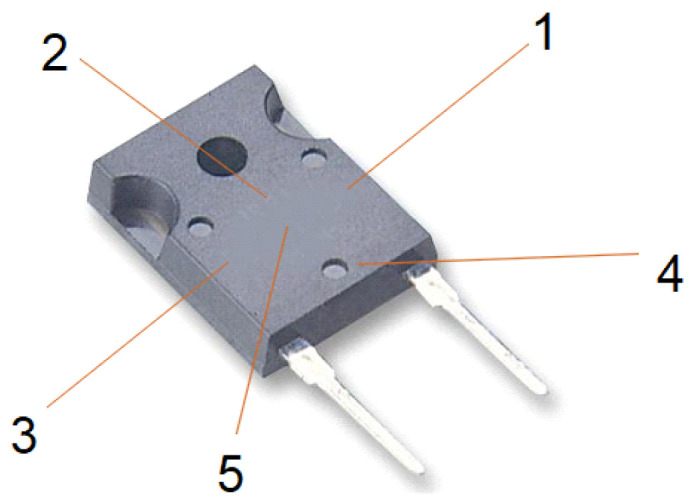
The location of the selected points on the mold body, the black part of the diode case made of epoxy resin (1–4) and the numerically designated place of the hottest spot on mold body 5. Point 5 was placed in the center of the lower part of the mold body (area below the circular holes).

Using LabVIEW 2022 software, the hottest point on the mold body was numerically determined for each *P_j_* value. In addition, four points were selected for the mold body, located at the edges. The location of the selected points and the numerically determined hottest point on the mold body are shown in Figure 18.

The temperature values obtained on the basis of thermographic *T_cT_* measurements in the selected places and in the numerically designated warmest place were compared with the temperature values obtained at the same points of the mold body based on simulations in SolidWorks *T_cS_*. The values obtained are presented in Table 4.

For each of the selected points, the die *T_jS_* temperature and the temperature differences between the *T_cS_* case temperature, determined by the simulation, and the *T_jS_* die temperature values determined on the basis of these simulations, were determined on the basis of the simulations. The results obtained are presented in Table 5.

## 4. Discussion

The size of the case, the mold body and the internal dimensions of semiconductor elements depend on the type of the case. The ratio of the size of the case to the size of the mold body differs too. However, the external dimensions of the case are standardized. Therefore, the temperature gradient between two points of a semiconductor element (located vertically or horizontally, relative to each other) depends on the location of these points and the distance between them. For different types of cases, the temperature differences between the case and the surface of the mold body on which the die is placed, determined in different places in this case, are different from each other. A smaller value of this difference allows for the estimation of the die temperature value with greater accuracy. Therefore, it was necessary to find an area on the surface of the mold body where the recorded temperature would have the highest values.

In the work carried out, the Optris Xi400 thermographic camera was used. For this model, it is possible to record thermograms with a maximum frequency of *f* = 80 Hz. When the temperature distribution of the recorded thermograms is analyzed using LabVIEW 2022 software, it is possible to automatically find the warmest area of the mold body of the diode, in which the temperature is closest to the temperature of the die. The results of this work were confirmed by the simulation works carried out using SolidWorks software. Both the simulation work and the algorithm developed in LabVIEW determined the same area for the mold body of the diode.

## 5. Conclusions

During thermographic measurements of semiconductor elements, the size of the element is a significant obstacle. The mold bodies commonly used today are small in size, making thermographic measurement of the temperature of such a surface problematic. The greater the distance between the mold body and the lens of the thermographic camera, the more difficult it is to perform such a measurement.

In practice, the place on the diode mold body is more important. The temperature of a place, which is above the die, may vary more than the temperature of another place on the diode mold body, and the variation may be greater than 20 °C. The differences increase when power dissipation in the die increases (Table 5). The die temperature estimated on the basis of such a thermographic measurement (in a place on the diode mold body, which is not above the die) is burdened by a significant error.

When the tests were conducted, it was also noticed that the temperature distribution on the surface of the mold body of the semiconductor element was heterogeneous. Therefore, it can be assumed that the temperature distribution on the surface of the die is also heterogeneous. This is related, among others, to the heterogeneity of heat propagation and the irregularity of the *ε* value. The die is not located under the entire surface of the mold body, as a result of which different points on the case of the semiconductor device may have different temperatures. To minimize the error of die temperature estimation based on the temperature distribution of the mold body recorded using a thermographic camera, the area with the highest temperature should be found in the thermogram.

Manual determination of such an area can result in an incorrect assessment of the warmest area, especially when it is small in size. For this reason, a method has been proposed to automate this activity. The use of LabVIEW software enables the precise and automatic determination of the case area with the highest temperature shown on the recorded thermogram. As a result, the difference between the mold body temperature and the die temperature is the smallest (see Table 4 and Table 5). Therefore, the error in estimating the die temperature on the basis of an indirect thermographic measurement is thus minimized. The proposed method allows us to obtain repeatable results. The results of the work carried out confirmed the simulation work carried out on the model of the tested element, for the same values of power *P_j_* dissipated on the die as in the thermographic measurements. The results obtained might be used in the uncertainty budget of the indirect thermographic temperature measurement of the semiconductor die.

On the basis of the conducted works, it was found that the smallest differences between the die temperature and the case temperature occur at point 5 for the model shown in Figure 18, and this is a spot on the mold body surface above the die. In the remaining points, the differences are greater, with the largest ones in point 4 of this model. The proposed method makes it possible to automate the determination of the area on the case surface of a semiconductor element where the temperature recorded by thermography is the highest. This method can be particularly useful when assessing the temperature of semiconductors based on thermographic measurements of moving elements. In this case, the time to determine the area with the highest temperature is the shortest, and the precision of this determination the highest.

The presented results are the outcome of research on temperature differences between the part of the diode mold body above the die and the diode mold body above the base plate. This information was difficult to find for FFSH 10120A. The result was obtained for one diode case and was confirmed for three other cases. The consequence was a necessity to find a method that allowed us to obtain this information.

This method can be used with other cases of the TO case family. The works carried out are an introduction to further work, as a result of which, a methodology for the remote, indirect thermographic measurement of the die temperature of a semiconductor element will be developed too. The developed method will speed up the diagnostic process and help verify the selection of the cooling system. The result of further work will also be a miniaturized measurement system for real-time integration. A diode in a TO-220-2 case was used in the work carried out. It is one of many commonly used cases. For this reason, it is necessary to conduct further research, which will allow the relationship between the temperature of the mold body and the die temperature of those cases to be determined, as presented in this article. These works will expand the area of application of the proposed method.

In this research, a cooling system was used based on natural conditions. This type of cooling system may not be sufficient in all cases (e.g., for high-power electronics). Forced convection cooling systems are more efficient. These systems require electricity to operate. In the future, there may possibly be a problem with the delivery of cheap electrical energy from ecological sources. It is necessary to reduce electricity consumption. For this reason, it is interesting to investigate the relationship between the consumption of electrical energy and the efficiency of cooling systems.

## Figures and Tables

**Table 1:** Natural convection correlation coefficients [41]; *a_lam_* is the value of coefficient a for laminar flow, *b_lam_* is the value of coefficient *b* for laminar flow, *a_turb_* is the value of coefficient *a* for turbulent flow, and *b_turb_* is the value of coefficient *b* for turbulent flow.

Shape	Gr∙Pr	a_lam_	b_lam_	a_turb_	b_turb_
Vertical flat wall	10^9^	0.59	0.25	0.129	0.33
Upper flat wall	10^8^	0.54	0.25	0.14	0.33
Lower flat wall	10^5^	0.25	0.25	NA	NA

**Table 2:** The power *P_j_* values dissipated in the die, the current flowing through the *I_D_* junction, the measurement error of the current flowing through the *ΔI_D_* junction, the voltage at the diode terminals *V_D_*, the voltage measurement error at the diode terminals *ΔV_D_*, the value of *ε* mold body and the value of the convection coefficient *h_c_*.

P_j_ (W)	I_D_ (A)	ΔI_D_ (A)	V_D_ (V)	ΔV_D_ (V)	ΔP (W)	ε (−)	h_c_ (−)
0.57	0.53	0.02	1.07	0.02	0.03	0.97	7,51
1.32	1.08	0.03	1.22	0.02	0.05	0.97	8.53
1.71	1.47	0.03	1.16	0.02	0.06	0.96	9.03
2.19	1.78	0.04	1.23	0.02	0.07	0.96	9.34
2.55	1.99	0.04	1.28	0.02	0.08	0.95	9.62
3.01	2.26	0.04	1.33	0.02	0.10	0.91	9.91

**Table 3:** The parameters related to the materials used to build the tested diode.

Fragment of the Case	k (W/m·K)	The Material (−)
Mold body	0.25	The EMC material
The back of the case	400.00	Copper
The die	150.00	Silicon carbide
Leads	400.00	Copper
The material under the die	400.00	Copper

**Table 4:** The thermography-measured case temperature values *T_cT_* and the simulation results obtained in SolidWorks *T_cS_* for each of the points are shown in Figure 18.

Point	1		2		3		4		5	
P_j_ (W)	T_cT_ (°C)	T_cS_ (°C)	T_cT_ (°C)	T_cS_ (°C)	T_cT_ (°C)	T_cS_ (°C)	T_cT_ (°C)	T_cS_ (°C)	T_cT_ (°C)	T_cS_ (°C)
0.57	43.5	44.1	44.8	46.0	43.7	44.3	41.2	41.3	45.7	47.2
1.32	66.4	67.1	69.2	69.5	67.1	67.3	60.0	60.6	72.1	72.5
1.71	81.3	80.6	85.4	86.6	80.8	81.9	71.5	71.2	91.5	90.6
2.19	95.0	94.5	96.6	97.3	92.9	94.6	82.1	81.9	104.8	103.6
2.55	102.8	103.3	109.8	109.1	103.7	104.7	91.6	91.8	114.5	115.0
3.01	113.6	114.2	117.2	118.1	115.3	116.7	97.7	97.2	125.4	125.0

**Table 5:** Die temperature *T_jS_* and the differences between *T_cS_* temperature and die *T_jS_* temperature determined on the basis of the simulation.

Point		1	2	3	4	5
P_j_ (W)	T_jS_ (°C)	T_jS_–T_cS_ (°C)	T_jS_–T_cS_ (°C)	T_jS_–T_cS_ (°C)	T_jS_–T_cS_ (°C)	T_jS_–T_cS_ (°C)
0.57	51.7	7.6	5.7	7.4	10.4	4.5
1.32	82.4	15.3	12.9	15.1	21.8	9.9
1.71	104.5	23.9	17.9	22.6	33.3	13.9
2.19	120.5	25.0	23.2	25.9	38.6	16.9
2.55	134.9	31.6	25.8	30.2	43.1	19.9
3.01	148.7	34.5	30.6	32.0	51.5	23.7

## Data Availability

Not applicable.
